# Prevalence and Risk Factors of Overweight and Obesity among Children Aged 6–59 Months in Cameroon: A Multistage, Stratified Cluster Sampling Nationwide Survey

**DOI:** 10.1371/journal.pone.0143215

**Published:** 2015-12-04

**Authors:** Sébastien Tchoubi, Joëlle Sobngwi-Tambekou, Jean Jacques N. Noubiap, Serra Lem Asangbeh, Benjamin Alexandre Nkoum, Eugene Sobngwi

**Affiliations:** 1 Ministry of Public Health, Yaoundé, Cameroon; 2 School of Health Sciences, Catholic University of Central Africa, Yaoundé, Cameroon; 3 Clinton Health Access Initiative, Yaoundé, Cameroon; 4 Department of Medicine, Groote Schuur Hospital and University of Cape Town, Cape Town, South Africa; 5 Medical Diagnostic Center, Yaoundé, Cameroon; 6 Department of Internal Medicine and Specialties, Faculty of Medicine and Biomedical Science, University of Yaoundé I, Yaoundé, Cameroon; 7 Laboratory for Molecular Medicine and Metabolism, Biotechnology Center, University of Yaoundé I, Yaoundé, Cameroon; 8 National Obesity Center, Yaoundé Central Hospital, Yaoundé, Cameroon; National Institute of Agronomic Research, FRANCE

## Abstract

**Background:**

Childhood obesity is one of the most serious public health challenges of the 21st century. The prevalence of overweight and obesity among children (<5 years) in Cameroon, based on weight-for-height index, has doubled between 1991 and 2006. This study aimed to determine the prevalence and risk factors of overweight and obesity among children aged 6 months to 5 years in Cameroon in 2011.

**Methods:**

Four thousand five hundred and eighteen children (2205 boys and 2313 girls) aged between 6 to 59 months were sampled in the 2011 Demographic Health Survey (DHS) database. Body Mass Index (BMI) z-scores based on WHO 2006 reference population was chosen to estimate overweight (BMI z-score > 2) and obesity (BMI for age > 3). Regression analyses were performed to investigate risk factors of overweight/obesity.

**Results:**

The prevalence of overweight and obesity was 8% (1.7% for obesity alone). Boys were more affected by overweight than girls with a prevalence of 9.7% and 6.4% respectively. The highest prevalence of overweight was observed in the Grassfield area (including people living in West and North-West regions) (15.3%). Factors that were independently associated with overweight and obesity included: having overweight mother (adjusted odds ratio (aOR) = 1.51; 95% CI 1.15 to 1.97) and obese mother (aOR = 2.19; 95% CI = 155 to 3.07), compared to having normal weight mother; high birth weight (aOR = 1.69; 95% CI 1.24 to 2.28) compared to normal birth weight; male gender (aOR = 1.56; 95% CI 1.24 to 1.95); low birth rank (aOR = 1.35; 95% CI 1.06 to 1.72); being aged between 13–24 months (aOR = 1.81; 95% CI = 1.21 to 2.66) and 25–36 months (aOR = 2.79; 95% CI 1.93 to 4.13) compared to being aged 45 to 49 months; living in the grassfield area (aOR = 2.65; 95% CI = 1.87 to 3.79) compared to living in Forest area. Muslim appeared as a protective factor (aOR = 0.67; 95% CI 0.46 to 0.95).compared to Christian religion.

**Conclusion:**

This study underlines a high prevalence of early childhood overweight with significant disparities between ecological areas of Cameroon. Risk factors of overweight included high maternal BMI, high birth weight, male gender, low birth rank, aged between 13–36 months, and living in the Grassfield area while being Muslim appeared as a protective factor. Preventive strategies should be strengthened especially in Grassfield areas and should focus on sensitization campaigns to reduce overweight and obesity in mothers and on reinforcement of measures such as surveillance of weight gain during antenatal consultation and clinical follow-up of children with high birth weight. Meanwhile, further studies including nutritional characteristics are of great interest to understand the association with religion, child age and ecological area in this age group, and will help in refining preventive strategies against childhood overweight and obesity in Cameroon.

## Introduction

Obesity is a major public health problem worldwide owing to its high prevalence and consequential morbidity and mortality [1,2]. Obesity is associated with cardiovascular, metabolic, psychological complications and risk of premature mortality in adulthood [2–4]. Globally, 44% of diabetes, 7% of ischemic heart disease and 41% of certain cancers are attributable to overweight and obesity [1]. In children, although morbid disorders appear only in case of severe obesity [5], obesity is also associated with psychological problems like negative emotions low self-esteem, and body image disparagement [6]. One of the major concern is that childhood obesity is likely to persist into adolescence and to adulthood [7]

Results from a meta-analysis based on 450 nationally representative surveys from 144 countries, showed that the overall prevalence of overweight and obesity increased from 4.2% in 1990 to 6.7% in 2010 among preschool children [8]. According to WHO, almost 42 million children aged less than five years (< 5 years) were obese or overweight in 2013 [1].

Childhood overweight and obesity has become a matter of great concern in developing countries. For example in Africa, 8.5% of preschool children were overweight during the same year and it is estimated that 12.7% of children (<5 years) will be affected in Africa by 2020 [8].

In few cases, obesity is caused by some genetic abnormalities [9]. Obesity can be attributed to certain medical causes such as hypothyroidism, hormonal growth deficiency or side effects of certain medications such as steroids [10]. However, several factors acting at different periods of growth are associated with overweight and obesity in children [11]. Socioeconomic status, level of education, marital status and BMI of parents, maternal smoking during pregnancy, sex, birth weight and the child's birth rank [7,12–14], area of residence [15,16] and some nutritional factors such as early introduction (<6 months of age) of solid foods and fruits [14,17] have been found as predictors of childhood overweight and obesity. Tabacchi et al [11] suggested in their literature review and a new classification of the early determinants of childhood obesity that religion can influence food behaviors through the definition of rules that identify the group, making it different from the others. Otherwise, it can influence eating style and consequently weight status.

In Cameroon, a low-income country which is still scarred by childhood malnutrition, childhood obesity is not yet perceived as an emerging health issue and receives little attention. A 2013 WHO report [18] based on data from previous Cameroon Demographic Health Surveys (DHS) showed that the overall prevalence of overweight (weight for height > 2SD) in children less than 5 years old had more than doubled between 1991 and 2006, from 4.7% to 9.6% [18]. To the best of our knowledge, no study to date has focused on factors associated with risk of overweight/obesity among children less than 5 years in Cameroon. This study sought to determine the prevalence of early childhood overweight/obesity and identify risk factors in Cameroon, using a representative sample of children aged 6 to 59 months from the DHS 2011. Findings will contribute to the design of effective preventive strategies to tackle the rising burden of early childhood overweight and obesity and its consequential morbidity and mortality in adulthood in Cameroon.

## Methods

### Ethics statement

The current study is based on publicly available data from the fourth Demographic Health Survey Cameroon Database. This Demographic Health Survey fulfilled all ethical requirements and was approved by the Cameroon Ministry of Public Health. Participants’ records in the database are anonymized, thus we did not have access to the identity of participants. The retrospective use of these data was approved by the Institutional Review Board of the School of Health Sciences, Catholic University of Central Africa, Yaoundé, Cameroon.

### Type of study and participants

DHS provides data from representative cross-sectional studies that are carried out in the general population, using validated questionnaires. DHS surveys are regularly implemented in over 50 countries worldwide and permit for the main basic demographic indicators and the health situation in those countries to be estimated and updated.

In Cameroon, the fourth DHS was conducted from January to August 2011 together with the "Multiple Indicator Cluster Survey" (MICS). Three standard questionnaires (women, men and household) were used [19]. A stratified national sample of 15,050 households was selected randomly in two stages: 580 clusters (or enumeration areas) were drawn for the first stage which included 291 and 289 clusters from urban and rural areas respectively. A count within each cluster led to a list of households from which was conducted a systematic sampling with equal probability. The final survey was carried out in 578 clusters and had included a national representative sample of 11,732 children less than five years from 14,214 households. Eligible households for the mothers’ and children’s anthropometric measurements (weight and height) were chosen by a systematic random draw conducted amongst selected household for the final survey (one household out of two) [19], which resulted to merely 50% of children in the database who had their weight and height recorded and their BMI z-score calculated.

We used the kids file dataset of the 2011 DHS database. we excluded children aged less than 6 months since some interventional studies that used an intensive, multidisciplinary approach or parental coaching demonstrated a significant decrease of adiposity over 6 months or at 6 months of age [20]. Were also excluded those whose BMI z-score were missing or was recorded in the database as “Height out of plausible limits” or “Age in days out of plausible limits” or “Flagged cases”, as their values were unusable since they were recorded in the database under special codes which corresponded either to responses that were considered inconsistent with other response in the questionnaire and thought to be probably an error, or to responses which value was “Don’t know”[21]. Finally, a total of 4518 children aged 6–59 months including 2313 girls and 2205 boys living with their mothers were included in our analysis.

The authorization for the access to the whole DHS database were obtained after a request explaining what we were intending to do with and which we sent through DHS program website at the address https://dhsprogram.com/

### Overweight and obesity

The BMI z-score based on WHO 2006 reference population [22] was used to assess overweight and obesity among children. The thresholds were defined according to WHO recommendations [23]: children who had a BMI z-score less than -2 were considered as thin; those who had a BMI z-score ranging from -2 to 2 were classified as normal weight children; those who had a BMI z-score over 2 were classified as overweight and children who had a BMI z-score greater than 3 were considered as obese. We excluded any BMI z-score recorded under the categories “Height out of plausible limits”, “Age in days out of plausible limits”, “Flagged cases”.

### Independent variables

Our independent variables were identified using the standard questionnaires and the DHS recoding manual (version 1.0, August 2012) [21]. Children, mothers’ and household characteristics that we considered to be relevant based on literature were chosen amongst the existing variables in the database. A minimal dataset was obtained after having applied exclusion criteria on BMI. We considered as “missing”, any missing case or any case that was recorded in the dataset as “Flagged”.

#### Maternal characteristics

Maternal BMI, initially a quantitative variable was recoded into four classes, based on the international classification of adult overweight and obesity [24]: thin (<18.5kg/m^2^), normal weight (18.5–24.9kg/m^2^), overweight (25–29.9kg/m^2^) and obese (≥30kg/m^2^). Cases that were recorded as ‘Flagged’ were classified as missing. Implausibility of maternal BMI values was checked using BMI<11 or >70kg/m^2^ as threshold of exclusion according a Canadian study [25], since any standard cut off point were found in Sub-Saharan studies. Highest level of education comprised four categories: had never been to school, primary, secondary and higher education. Mothers’ religion was regrouped into Christian (catholic and protestant), animist, Muslim, or other religions categories. For mother’s marital status, the initial classes "married” and “cohabiting with a partner" were regrouped into "couple" category; the other initial classes (“single”, “widowed”, “divorced” and “separated”) were put together in “single parent" category. Mother’s occupation derived from Respondent currently working variable and comprised two classes: currently employed and not employed. Type of place of residence was urban or rural.

#### Household characteristics

Wealth index of household, a variable indicating an economic well being score based on housing characteristics and ownership of sustainable goods [26], initially comprised fives levels: poorest, poorer, middle, richer, richest. Given some similarities between “poorest” and “poorer”, and between “richer” and richest”, the first two classes were aggregated and renamed “low economic status” and the last two classes were also merged and renamed “high economic status”. The class “middle” was kept as initially. Number of children under 5 years in the household was broken into two classes based on its gross distribution of frequency: 1–2 children and >2 children

#### Child characteristics

Age was recoded in 12 months groups: 6–12, 13–24, 25–36, 37–48, and 49–59 months. Birth weight was categorized into three groups, based on clinical cut off points and WHO definition of low birth weight: low (<2500g), middle (2500g-4000g) and high birth weight (>4000g). As we did not have any data on gestational age in the dataset to deal with implausible birth weight values according to one of the current rules of exclusion [27], all birth weight > 5500g or < 900g were deemed unlikely to be valid since they were considered clinically implausible. Any case which was originally recorded as ‘not weighted’ or ‘Don’t know ‘was classified in the missing group. Based on gross distribution of frequency, Child’s birth rank derived from ‘Birth order variable’, which was cut into two classes: low birth rank (from 1 to 3) and high birth rank (>3). The variable “region” comprised 12 categories, corresponding to the 10 administrative regions of Cameroon except “Douala” and “Yaoundé” which are the two greatest urban cities, respectively the economic and the political capital, and which belong to Littoral and Center region respectively. Each of these regions is considered as a study area according to DHS survey plan. As regions may be grouped according to their lifestyle and other geographical considerations, this variable was recoded in four ecological areas: Savannah area (Far North, North and Adamawa Regions), Forest area (Centre East and South Regions), Coastal area (Littoral, South-West Regions) and Grassfield area (North-West, West Regions).We kept “Douala” and “Yaoundé” in the new variable since they are the most urbanized and cosmopolite settings in Cameroon.

### Statistical analyses

Variables of interest were selected using the Software Package for Statistics and Simulation SPSS version 16.0 for Windows (SPSS, Chicago, Illinois, USA), since DHS databases exist in SPSS format. Data were imported into R software (version 3.1.3) for analysis. A univariate descriptive analysis was performed to assess the structure of all selected variables and to estimate the overall proportions of obese, overweight, normal weight and thin children. A descriptive bivariate analysis was used to examine disparities in overweight and obesity prevalence pertaining to ecological areas, sex and type of place of residence. A Chi square test or eventually a Fisher test and Student t test were used to compare mothers, children and households characteristics amongst thin, normal weight and overweight/obese children. Birth weight, mother religion, Maternal BMI and occupation had respectively almost 40%, 0.5%, 0.4% and 0.2% of missing values, thus we looked at bias issue focusing our interest on birth weight as this variable had the highest and a huge percent of missing values. We found that almost 88% of children with missing value in birth weight had normal weight. Since normal weight children were more likely to have normal birth weight, we assumed that birth weight might been missing in children who were more likely to have normal birth weight and we made the missing at random assumption as this explanation of missing data emerged from the dataset. Thus, imputation of missing data was needed to prevent bias associated with birth weight missing data. Also, we observed that children with birth weight missing values were more likely to live in Savannah area (67%), in rural setting (81%) and in households with low economic status (almost 71%) and to have mothers with lowest level of education (91% of none and primary as the highest level). In addition, children whom mother had lower level of education were more likely to live in savannah area, in rural setting and in households with low economic status. Finally, children whom mother had lover level of education or who lived in rural settings or in household of low economic status were less likely to be obese and overweight, Consequently, to be consistent with the plausibility of the missing at random assumption [28], all these covariates and our outcome variable as well as all other variables were fitted into our imputation model. Data on maternal BMI and religion were also imputed using the same model although these variables relatively had small amount of missing data. Data were imputed using the imputation method of incomplete categorical datasets with a Multiple Correspondence Analysis model [29].

Also, we checked out multicolinearity issue between economic status, parent highest level of education and number of children less than five years in a household as they were a correlation between these variables and found no issue of multicolinearity between them (all their Variance Inflation Factors were less than 3 in multicolinearity test)

Odds ratios (OR) with their 95% confidence intervals (CI) served to investigate the factors associated with overweight and obesity. They were calculated by both univariate and multivariate logistic regression analyses while adjusting for potential confounders. We included in the multivariate model all variables with a *p* value ≤ 0.20 in univariate analyses with child’s age and sex and maternal BMI to be forced in the model even if above the selection limit. Thin children (n = 204) were excluded from the univariate and multivariate regression analysis since we were comparing normal weight to overweight and obese children. Children with birth weight > 5500g (n = 19) were also excluded. Estimates that were obtained from imputed data, both in univariate and multivariate regression analysis were compared to the results of complete case analysis to ascertain their consistency with what were assumed or expected. A *p* value < 0.05 was set as statistically significant.

## Results

### Description of the study population

Mean age of included children was 30.2 (SD 15.8) months. Girls made up 51.2% of the study population, and the majority of children were recruited from in rural areas (58.5%). Children's birth weight ranged from 900 to 9500 grams with 19 children (0.4%) having a birth weight more than 5500g. Number of children (< 5 years) in households ranged from one to 19, with a mean of 2 (SD 1) children per household. Birth rank ranged from one to 15 and almost 57% of children belonged to the first to third birth rank category (Table 1). Maternal BMI ranged from 13.3 to 56.9 kg/m^2^ with a mean BMI of 23.8 (SD 4.6) kg/m^2^. BMI z-scores varied from -4.9 to 4.7 with a mean z-score of 0.27 (SD 1.33). Eighty seven point five percent (3951/4518) of children had normal weight, 8% (363/4518) were overweight of whom 1.7% were obese (75/4518) and 4.5% (204/4518) were thin (Table 2). The prevalence of overweight and obesity (together) in urban areas was 8.7% (1.9% for obesity alone) compared to 7.6% (1.5% for obesity alone) in rural areas.

**Table 1:** Children characteristics among thin, normal weight and overweight children aged 6 to 59 months, Cameroon, 2011.

Characteristics		Weight status		
	Total (N = 4518)n (%)	Thin (n = 204)**n (%)**	Normal weight (n = 3951)**n (%)**	Overweight/obese (n = 363)**n (%)**
**Ecological area**				71 (19.6)
Savannah	1616 (35.8)	137 (67.2)	1408 (35.6)	
Forest	927 (20.5)	42 (20.6)	831 (21)	54 (14.9)
Coastal	524 (11.6)	7 (3.4)	467 (11.8)	50 (13.8)
Grassfield	856 (18.9)	10 (4.9)	715 (18.1)	131 (36.1)
Douala	324 (7.2)	5 (2.5)	287 (7.3)	32 (8.8)
Yaoundé	271 (6)	3 (1.5)	243 (6.2)	25 (6.9)
**Area of residence**				
Urban	1900 (42.1)	49 (24)	1686 (42.7)	165 (45.5)
Rural	2618 (57.9)	155 (76)	2265 (57.3)	198 (54.5)
**Child’s age (in months)**				
0–12	756 (16.7)	79 (38.7)	632 (16)	45 (12.4)
13–24	1118 (24.7)	67 (32.8)	959 (24.3)	92 (25.3)
25–36	963 (21.3)	26 (12.7)	815 (20.6)	122 (33.6)
7–48	907 (20.1)	15 (7.4)	828 (21)	64 (17.6)
49–59	774 (17.1)	17 (8.3)	717 (18.1)	40 (11)
**Birth weight (in grams)**				
< 2500	210 (4.6)	11 (5.4)	184 (4.7)	15 (4.1)
2500–4000	2114 (46.8)	48 (23.5)	1862 (47.1)	204 (56.2)
4000–5500	462 (10.2)	12 (5.9)	383 (9.7)	67 (18.5)
>5500	19(0.4)	0(0)	16(0.4)	3(0.8)
Missing	1713 (37.9)	133 (65.2)	1506 (38.1)	74 (20.4)
**Sex**				
Male	2205 (48.8)	117 (57.4)	1874 (47.4)	214 (59)
Female	2313 (51.2)	87 (42.6)	2077 (52.6)	149 (41)
**Birth rank**				
1–3	2573 (56.9)	99 (48.5)	2246 (56.8)	228 (62.8)
>3	1945 (43.1)	105 (51.5)	1705 (43.2)	135 (37.2)

Data are presented as frequency (percentage)

**Table 2:** Mother and Household characteristics among overweight, obese and thin children aged 6 to 59 months, Cameroon, 2011.

Characteristics		Weight status		
	Total (N = 4518) n (%)	Thin (n = 204) n (%)	Normal weight (n = 3951) n (%)	Overweight/obese (n = 363) n (%)
**Mother characteristics**				
**Mother’s age (years**)				
15–25	1770 (39.2)	98 (48)	1530 (38.7)	142 (39.1)
25–35	2031 (45)	86 (42.2)	1788 (45.3)	157 (43.3)
**>**35	717 (15.9)	20 (9.8)	633 (16)	64 (17.6)
**Marital status**				
Single parent	492 (10.9)	17 (8.3)	430 (10.9)	45 (12.9)
Parents living together	4026 (89.1)	187 (91.7)	3521 (89.1)	318 (87.6)
**Maternal BMI (kg/m** ^**2**^ **)**				
< 18.5	311 (6.9)	37 (18.1)	266 (6.7)	8 (2.2)
18.5–24.9	2775 (61.4)	137 (67.2)	2458 (62.2)	180 (49.6)
25–29.9	981 (21.7)	23 (11.3)	848(21.5)	110 (30.3)
≥30	434 (9.6)	6 (3.4)	362 (9.2)	65 (17.9
Missing	17 (0.4)	0 (0)	17 (0.4)	0 (0)
**Level of education**				
None	1012 (22.4)	94 (46.1)	881 (22.3)	37 (10.2)
Primary	1997 (44.2)	71 (34.8)	1744 (44.1)	182 (50.1)
Secondary	1372 (30.4)	37 (18.1)	1209 (30.6)	126 (34.7)
Higher	137 (3)	2 (1)	117 (3)	18 (5)
**Religion**				
Christian	3214 (71.1)	118 (57.8)	2797 (70.8)	299 (82.4)
Muslim	1020 (22.6)	69 (33.8)	901 (22.8)	50 (13.8)
Animist	135 (3)	9 (4.4)	122 (3.1)	4 (1.1)
Other	127 (2.8)	6 (2.9)	112 (2.8)	9 (2.5)
Missing	22 (0.5)	2 (1)	19 (0.5)	1 (0.3)
**Occupation**				
Unemployed	1308 (29)	74 (36.3)	1150 (29.1)	84 (23.1)
Employed	3201 (70.8)	130 (63.7)	2794 (70.7)	277 (76.3)
Missing	9 (0.2)	0 (0)	7 (0.2)	2 (0.6)
**Household’s characteristics**				
**Economic status**				
Low	1935 (42.8)	138 (67.6)	1666 (42.2)	131 (36.1)
Middle	1611 (35.7)	31 (15.2)	1438 (36.4)	142 (39.1)
High	972(21.5)	35 (17.2)	847 (21.4)	90 (24.8)
**Number of children (< 5years)**				
1–2	2900 (64.5)	113 (55.4)	2525 (63.9)	262 (72.2)
>2	1618 (35.8)	91 (44.6)	1426 (36.1)	101 (27.8)

**Fig 1:**
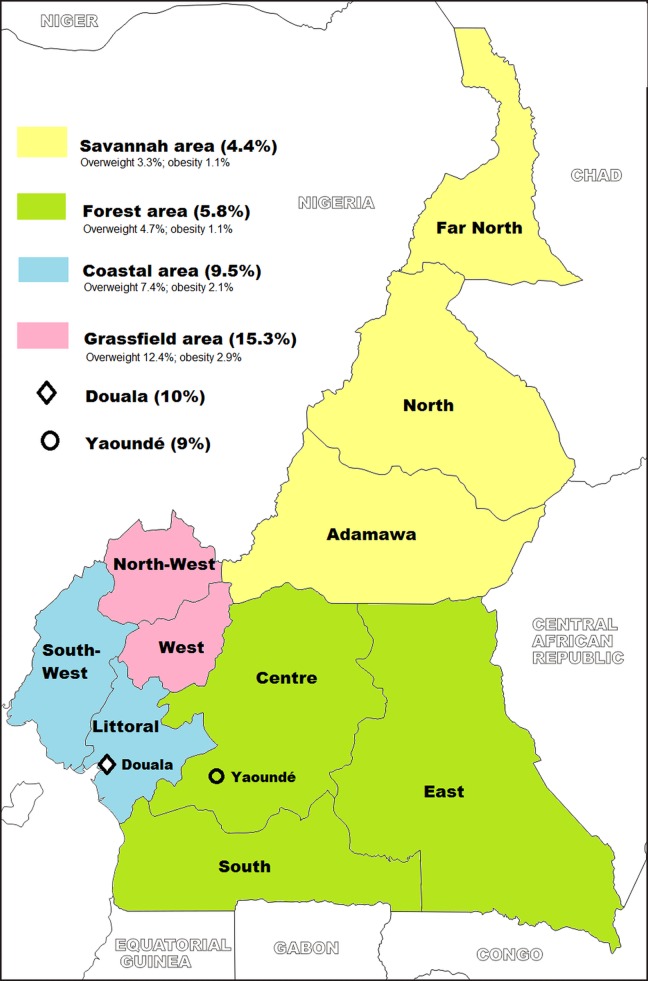
National distribution of prevalence rates of overweight and obesity among children aged 6 to 59 months, according to ecological areas, Cameroon, 2011.

Boys were more likely to be overweight and obese than girls with a prevalence of 9.7% and 6.4% respectively. Grassfield areas had the highest prevalence of overweight and obesity (15.3%), followed by Coastal areas (9.5%), while the lowest prevalence was found in Savannah (4.4%) and Forest areas (5.8%). Nearly 10% and 9% of children were overweight or obese in Douala and Yaoundé respectively. Globally, we found a North-South and an East-West ascending gradient in childhood overweight and obesity distribution (Fig 1).

### Risk factors for overweight/obesity

Results from multivariate logistic regression (Table 3) showed that having overweight and obese mother compared to having normal weight mother was significantly associated with a higher risk of overweight (adjusted odd ratio (aOR) = 1.51; 95% CI 1.15 to 1.97.) and (aOR = 2.19; 95% CI 155 to 3.2) respectively, as well as high birth weight (>4000g), compared to average (2500–4000g) birth weight (aOR = 1.69; 95% CI 1.24 to 2.28), male gender (aOR = 1.56; 95% CI 1.24 to 1.95), low birth rank (1 to 3), compared to high birth rank(> 3), (aOR = 1.35; 95% CI 1.06 to 1.72). Also, children’s age was independently associated with overweight. Compared to children aged 49 to 59 months, an increasing risk was found with children aged 13 to 36 months (Table 2): 13 to 24 months (aOR = 1.78; 95% CI = 1.21 to 2.66), and 25 to 36 months (aOR = 2.79; 95% CI 1.93 to 4.13). The association with age was also positive with 6 to 12 and 37 to 45 months age group but was not significant (aOR = 1.32; 95% CI = 0.84 to 2.08) and (aOR = 1.45; 95% CI 0.96 to 2.22) respectively. Ecological area was also found to be independently associated with overweight; living in the Grassfield areas being associated with a higher risk of overweight (aOR = 2.65; 95% CI = 1.87 to 3.79) compared to Forest areas. A protective effect was found with the Muslim religion (aOR = 0.67; 95% CI 0.46 to 0.95) compared to Christian religion.

On the other hand, residence, mother’s highest level of education and occupation, economic status, number of children <5 years in household were associated with being overweight but the associations did not remain significant in the multivariate analysis (Table 3). Also, no significant association was observed with marital status, mother’s age and residence (Table 3).

**Table 3:** Univariate and multivariate analysis showing factors independently associated with overweight/obesity in children aged 6 to 59 months, Cameroon, 2011.

Characteristics	Unadjusted model		Adjusted model^a^	
	OR (95% CI)	P-value	OR (95% CI)	P-value
**Mothers characteristics**				
**Maternal BMI (kg/m** ^**2**^ **)**		<0.001		<0.001
< 18.5	0.41 (0.19–0.80)	0.02	0.55 (0.24–1.07)	0.10
18.5–24.9	1		1	-
25–29.9	1.78 (1.38–2.28)	<0.001	1.51 (1.15–1.97)	0.002
≥30	2.44 (1.79–3.30)	<0.001	2.19 (1.55–3.07)	<0.001
**Age**		0.61	-	-
15–25	1.06 (0.84–1.35)	0;65	-	-
26–35	1	-	-	-
>35	1.17 (0.85–1.57)	0.37	-	-
**Marital status**		0.38	-	-
Living single	1.16 (0.83–160)	0.38	-	-
Living in couple	1		-	-
**Highest level of education**		<0.001		0.53
None	1		1	-
Primary	2.48 (1.75–3.62)	<0.001	1.29 (0.84–2.02)	0.25
Secondary	2.44 (1.69–3.61)	<0.001	1.27 (0.78–2.09)	0.35
Higher	3.65 (1.98–6.55)	<0.001	1.65 (0.80–3.36)	0.17
**Religion**		<0.001		0.04
Christian	1		1	-
Muslim	0.51 (0.37–0.69)	<0.001	0.67 (0.46–0.95)	0.03
Animist	0.31 (0.09–0.74)	0.02	0.41 (0.12–1.02)	0.09
Other	0.76 (0.35–1.42)	0.42	0.74 (0.34–1.45)	0.42
**Occupation**		0.01		0.3
Unemployed	0.73 (0.56–0.94)	0.01	0.89 (0.67–1.16)	0.38
Employed	1		1	-
**Household’s characteristics**				
**Economic status**		0.06		0.29
Low	1	-	1	-
Middle	1.25 (0.98–1.61)	0.08	0.81 (0.57–1.12)	0.19
High	1.37 (1.03–1.81)	0.03	1.00 (0.75–1.40)	0.86
**Number of children (< 5years)**		0.002		0.10
1–2	1.45 (1.14–1.84)	0.001	1.25 (0.96–1.58)	0.09
>2	1		1	-
**Children characteristics**				
**Ecological area**		<0.001		<0.001
Savannah	0.78 (0.54–1.12)	0.17	1.31 (0.86–2.00)	0.21
Forest	1	-	1	-
Coastal	1.64 (1.10–2.46)	0.01	1.42 (0.94–2.15)	0.09
Grassfield	2.79 (2.01–3.91)	<0.001	2.65 (1.87–3.79)	<0.001
Douala	1.71 (1.07–2.69)	0.02	1.54 (0.93–2.52)	0.09
Yaoundé	1.54 (0.91–2.51)	0.09	1.42 (0.82–2.40)	0.20
**Place of residence**		0.33		
Urban	1.11 (0.90–1.38)	0.31	-	-
Rural	1		-	-
**Age (in months)**		<0.001		<0.001
6–12	1.25 (0.81–1.95)	0.31	1.33 (0.84–2.08)	0.22
13–24	1.68 (1.15–2.49)	0.008	1.81 (1.21–2.66)	0.004
25–36	2.69 (1.87–3.93)	<0.001	2.79 (1.93–4.13)	<0.001
37–48	1.38 (0.92–2.09)	0.12	1.46 (0.96–2.22)	0.08
49–59	1	-	1	
**Birth weight (in grams)**		<0.001		0.002
< 2500	0.99 (0.55–1.64)	0.96	0.83 (0.46–1.41)	0.51
2500–4000	1	-	1	-
> 4000	2.12 (1.58–2.81)	<0.001	1.69 (1.24–2.28)	0.0004
**Sex**		<0.001		0.0001
Male	1.59 (1.28–1.99)	<0.001	1.56 (1.24–1.95)	0.0001
Female	1	-	1	-
**Birth rank**		0.04		0.01
1–3	1.26 (1.01–1.58)	0.03	1.35 (1.06–1.72)	0.01
> 3	1		1	-

OR, odd ratio; CI, confidence interval

^**a**^ values based on a logistic regression model including maternal characteristics (BMI, level of education, region of residence, area of residence, mother’s religion), child’s characteristics (birth weight, sex), and household characteristics (number of children (< 5years) in the household, family economic status). Thin children were excluded from the analysis, missing data imputed.

## Discussion

This study sought to determine the prevalence of overweight and obesity and identify risk factors among children aged 6 to 59 months in Cameroon using DHS-MICS, 2011 data. Our findings showed that in 2011, the overall prevalence of joined overweight and obesity (BMI z-score for age) in Cameroon was 8% (1.7% for obesity alone). Children who were living in Grassfield areas were the most affected by overweight and obesity. In addition, being a boy, being aged between 13 to 36 months and belonging to a lower birth rank category were independently associated with higher risk of overweight or obesity. Likewise, children whose mothers were overweight or obese were at higher risk of overweight/obesity. Muslim religion appeared to be slightly protective against overweight/obesity. A significant independent association was not found with highest level of education, economic status and mother's occupation. No significant association was found with residence, mother's age, and marital status

The main strength of this study is that we used a nationally representative sample of children aged 6 to 59 months, allowing generalization of the results. Furthermore, this study is one of the first that assesses factors associated with the risk of overweight/obesity and highlights disparities according to ecological area among children in Cameroon.

We found an overall prevalence of overweight and obesity that might be higher compared to estimates based on weight for height index. By comparing changes in the prevalence of overweight in preschool children between 1990 and 2010, De Onis et al. found that estimates using BMI z-score were always greater than those observed using weight for height z-score [8]. Furthermore, our findings suggest that Cameroon has one of the highest prevalence (8%) of overweight in children less than 5 years as compared to several other sub-Saharan African countries like Ivory Coast (4.4% in 2012), Nigeria (6.3% in 2013), Senegal (6.8% in 2013), Democratic Republic of Congo (5.6% in 2014) where relatively lower prevalence had been found [30].

Our univariate and multivariate logistic regression analysis from imputed data globally yielded similar results compared to estimates that were obtained from complete cases analysis. In return, the magnitude of birth weight estimates increased with imputed data, which was consistent with what was expected and assumed since there was an increasing in statistical power because of imputation. The imputation of maternal BMI and religion missing values did not change estimates with these variables since they had small amount of missing data.

This study showed that children living in Grassfield areas were more than two and half-fold at risk of overweight compared to their peers who lived in Forest areas. To our knowledge, our study is one of the first that demonstrate such a significant disparity across ecological areas as regards overweight in children. Our analysis showed a strong association between ecological area and overweight (p <0.001), which persisted even after adjusting all associated factors, suggesting that contribution of this factor to the development of overweight and obesity differs in magnitude from one ecological area to another, living in Grassfield area being more favorable for the development of overweight and obesity than living in any other ecological area. Differences in lifestyle, diet or in feeding habits across ecological areas may be worth investigating in such association. Even after adjustment, the strength of the association changed slightly with Grassfield areas showing that this area is independently associated with overweight and obesity. Meanwhile, the higher risk of overweight associated with Coastal areas and Douala did not remain significant, showing that living in Coastal areas was not an independent factor associated with overweight.

This study also underlined the gender difference in overweight with boys being more at risk of overweight than girls in of this age group in Cameroon. The relation between gender and overweight in children is inconsistent across literature with boy predominance in some studies [12,31,32], girl predominance [33–36], or no difference observed [7,37], supporting the hypothesis that overweight is a result of interactions between genetic and environmental factors [11].

Our study supported the important role played by birth weight in the emergence of overweight in Cameroon. Children with high birth weight were more at risk of overweight than children with normal birth weight. These findings are consistent with previous reports [14,17,38,39]. However, some studies have shown that low birth weight is associated with a higher risk of overweight or obesity, [33,40]. Indeed, many studies have shown that there is an association between smoking during the antenatal period and low birth weight, which generally leads to a rapid weight gain in the following six months; with a higher risk of overweight in children and adults [7,12]. Finally, intra-uterine conditions have been found to be associated with birth weight [41,42] which is associated to obesity at an early age.

This study also highlighted the role played by maternal BMI in the emergence of overweight. According to our findings, children whose mothers were overweight and obese were more at risk of overweight than those whose mothers were normal weight. Maternal BMI has been reported in previous studies to be a determinant of childhood overweight. High maternal BMI is known to be a strong predictor of high birth weight and of overweight in offspring [43,44]. Our study is consistent with a linear relationship between maternal BMI and early childhood obesity. Moreover, studies had reported that paternal BMI was also associated with overweight in children [12,45], supporting the hypothesis that parental BMI might determine overweight in children through genetic, environmental and behavioral mechanisms [11]

Also interesting was the relation that we found between age and overweight in children. Our study showed a significant gradual increase of the odds of being overweight from 13 to 36 months of age. To our knowledge, none of previous studies has not investigated such an association which might not suggesting a direct contribution of age for the development of early childhood overweight and obesity but might rather suggest these age groups as periods during what different factors to be investigated contribute more to the development of overweight and obesity in children. Infant feeding pattern at this period might be one of the factors of great interest to the understanding of such an association.

Birth rank emerged in this study as an overweight independently associated factor. Our results showed an inverse association with birth rank, children belonging to the category of first to third birth rank being more likely at risk of overweight than those of birth rank greater than three. Only few studies focused their interest on the relationship between birth rank and overweight in an early age. Dubois and Girard [12] showed in a longitudinal study among a representative sample of 2103 Canadian preschoolers that children of first rank were likely to have higher birth weight (> 4000g) but not to become overweight later at 4.5 years. Fatemeh et al [38] in a cross sectional study rather compared the prevalence of both birth ranks of 1 to 3 and of more than 3 between overweight and obese children aged 2 to 5 years old and did not find a significant difference. However a significant positive association of this factor with overweight has been found at later age in other cross sectional studies, children of the first positions being more at risk than those coming in later [46,47]. Our findings with birth rank are inconsistent with what had been reported in the former studies. Compared to our study, Dubois and Girard study involved many potential overweight risk factors that might have cancelled the expected effect of birth rank. In our analysis, the strength of this association remained constant after adjustment of other associated factors (Table 3), showing the stability of the association. However, investigations on birth rank in a cohort study is needed to ascertain the consistence of its effect on early childhood overweight and obesity.

The effect of religion is worth mentioning. Our multivariate analysis showed a weakly protective effect with Muslim religion compared to Christian religion. To our knowledge, such an association has not yet been reported in previous studies. However, according to Tabacchi et al [11], religion can influence eating behaviors through compliance with rules that distinguish religious groups. In other words, parent’s choice regarding child’s diet may be influenced by religion. This finding suggests that Muslim feeding habits or lifestyle might protect their children against overweight. Investigations to assess Muslim lifestyle, diet or feeding habits and its relationship with overweight are needed to understand such an effect. During our analysis, moving ecological area in our final model resulted in loss of significance of the association observed, showing that ecological area is a strong confounder in the association between religion and overweight. This confounding effect was expected since there was a North-South ascending gradient in childhood overweight distribution, given that Muslims live mostly in the Northern part of Cameroon.

More puzzling were our findings regarding economic status, mothers’ highest level of education and occupation. An inverse association with economic status, parental education, type of place of residence has been reported in developed countries [13,37,48] while in developing countries, this association has been found positive, children with high socioeconomic status, high parental education, living in urban settings have a higher risk of overweight [15]. Our findings are in line with what has been reported in developing countries, but the associations did not remain significant in multivariate analysis, showing that these factors were not independent. From our analysis, no significant association was found with type of place of residence, although overweight children lived more in urban areas. A way to reconcile these findings is that more children were recruited in rural than in urban settings, with a ratio of nearly 1.5, making the two groups incomparable and which may have biased the expected result towards absence of association with overweight.

This study did not show a significant association with mother’s age, marital status, in spite of the higher proportion of single parents and employed mothers among overweight children. Dubois and Girard [12] reported a positive association with married mothers before birth, also reported by Flores and Lin [17]. While Rooney et al.[7] and Huus et al [45] reported a positive association with single mothers before birth. Likewise, Mushtaq et al [49] and Fatemeh et al [38] found a positive association with employed mothers.

Our study has some limitations. Using an existing database limited us from including variables that did not initially exist. Nutritional characteristics such as child’s diet or feeding habits would have permitted us to assess their contribution to the development of overweight and get better adjusted estimates of associated factors. Moreover, since this study was cross-sectional, it has not been possible to know if certain characteristics like mother's age, occupation, marital status, preceded or followed child’s birth, which might have biased our estimates or our results towards absence of association. Also, because of this study design, it is impossible to either infer causability or untangle bi-directional relationships. One potential major limitation is the frequency of missing data especially concerning birth weight. We addressed this limitation by validated data imputation approaches to prevent the associated bias.

## Conclusion

This study points out a high prevalence of early childhood overweight with significant disparities between ecological areas of Cameroon. Risk factors of overweight included having overweight or obese mother, having high birth weight, being male gender, having low birth rank, being aged between 13–36 months, living in the Grassfield areas while being Muslim appeared as a protective factor. Preventive strategies should focus on sensitization campaigns to reduce overweight and obesity in parents and reinforcement of clinical measures such as weight gain surveillance during antenatal consultation which will help prevent children from high birth weight and overweight later in life. A clinical follow up of children with high birth weight is also necessary to prevent them from later childhood overweight. These campaigns and clinical measures should be especially strengthened in Grassfield area. Further studies including nutritional characteristics are of great interest to understand the association with religion, child age and ecological area in this age group. This will help in refining preventive strategies against childhood overweight and obesity in Cameroon and comparable or neighboring countries
